# Phlegmasia Dolens Occurring in a Female, and Not Connected with the Puerperal State

**Published:** 1852-09

**Authors:** John Kelly

**Affiliations:** Esperance, N. Y.


*Phlegmasia Dolens Occurring in a Female, and not Connected with the Puerperal State.* By John Kelly, M. D., of Esperance, N. Y.—(Communicated for the Boston Medical and Surgical Journal.) On the 7th of August, 1847, I was called to see Miss S. Scott, in the town of Schoharie, who had been many years feeble and rather leuco-phlegmatic. She was then laboring under fever, with headache, more on the left side of the head than on the right; pulse strong and tense. I thought it not advisable to bleed at this time, but a week after I bled her to the amount of about ℥ viij. This relieved the head, and perhaps a blister which I put to the nape of the neck had some good effect. The general treatment consisted in the use of blue pill, laxatives and digitalis purpura.

Aug. 23. Found her symptoms improving, headache gone, and appetite better; yet as her pulse was not quite soft enough to suit me, I thought best to continue treatment. In the absence of her mother about this time, she walked out to the orchard, and soon after complained of a pain in the hip near the joint.

Sept. 2. Found her left leg some swollen, thigh more so; the inside of thigh excessively tender to the touch, tense, veins enlarged, with rather dark streaks and some hard lumps. The whole limb perfectly useless, giving the sense to her of great weight. The pulse, at this time, was more strong and tense than ever. The tongue had a white coat. Ordered purgatives of Ep. salts and cream of tartar, with an occasional dose of chloride of mercury and febrifuges.

4th. Pain more intense than ever. Ordered anodynes and a powder of three fourths of a grain of digitalis and eight grains cream of tartar every four hours. Local applications of infusion of poppy and hot vinegar.

7th. Found her no better. Ordered sal. ammoniac dissolved, and laudanum to the most painful parts; and to be given every four hours, five grains cream of tartar, three fourths of a grain of opium, as she had not slept for three or four days and nights.

9th. Found her about the same. She wished to be moved often from her back to her side and vice versa. Ordered xxv. grs. calomel, crem. tart. v. grs., digitalis gr. j., every four or five hours.

10th. Calomel operated favorably. She was easier; gums slightly affected. The thigh not so much swollen, nor so tender. Ordered pill blue mass and digitalis daily, applying a wash of op. ʒ i., sal. ammoniac, ʒ jiss., camphor, ʒ jss., dissolved in spirits.

11th. Improving. Continue the same treatment.

12th. Improving. Continue same treatment, and a cathartic of cream of tartar and jalap.

A few days after the left leg had become better, the right one was also in the same way affected, though not so severely. The fever, which had subsided, came on again, and the same treatment had to be resorted to for the purpose of subduing the constitutional symptoms, which were not so severe as at the time the first leg was affected. For some ten days there was no great improvement, except the swelling subsided measurably; but the limb continued œdematous for some time, and extremely weak. Indeed she was not able to walk for six or seven weeks after she otherwise improved. Her pulse became more soft, and her appetite improved. Anodynes once or twice a-day, and a pill of socotrine aloes were continued for sometime to compose the nerves and to regulate the uterine system. After a short time her health became confirmed, and ever since she has been one of the most healthy young ladies to be found.—*Boston Medical and Surgical Journal.*

